# Distribution of transport injury and related risk behaviours in a large national cohort of Thai adults

**DOI:** 10.1016/j.aap.2010.12.011

**Published:** 2011-05

**Authors:** Karen Stephan, Matthew Kelly, Rod Mcclure, Sam-ang Seubsman, Vasoontara Yiengprugsawan, Christopher Bain, Adrian Sleigh, The Thai Cohort Study Team

**Affiliations:** a Monash University Accident Research Centre, Monash University, Melbourne, Australia; b National Centre for Epidemiology and Population Health, Australian National University, Canberra, Australia; c School of Human Ecology, Sukhothai Thammathirat Open University, Nonthaburi, Thailand; d The University of Queensland, School of Population Health, Brisbane, Australia

**Keywords:** Wounds and injuries, Accidents, Transportation, Thailand

## Abstract

**Background:**

A major barrier to addressing the problem of transport injury in low to middle-income countries is the lack of information regarding the incidence of traffic crashes and the demographic, behavioural and socio-economic determinants of crash-related injury. This study aimed to determine the baseline frequency and distribution of transport injury and the prevalence of various road safety behaviours in a newly recruited cohort of Thai adults.

**Methods:**

The Thai Health-Risk Transition Study includes an ongoing population-based cohort study of 87,134 adult students residing across Thailand. Baseline survey data from 2005 includes data on self-reported transport injury within the previous 12 months and demographic, behavioural and transportation factors that could be linked to Thailand's transport risks.

**Results:**

Overall, 7279 (8.4% or 8354 per 100,000) of respondents reported that their most serious injury in the 12 months prior to recruitment in the cohort was transport-related, with risk being higher for males and those aged 15–19 years. Most transport injuries occurred while using motorcycles. A much higher proportion of males reported driving after three or more glasses of alcohol at least once in the previous year compared to females. The prevalence of motorcycle helmet and seat belt wearing in this sample were higher than previously reported for Thailand.

**Conclusions:**

The reported data provide the basis for monitoring changes in traffic crash risks and risk behaviours in a cohort of adults in the context of ongoing implementation of policy and programs that are currently being introduced to address the problem of transport-related injury in Thailand.

## Research highlights

▶ 8.4% of Thai adults reported a transport related injury in the previous 12 months. ▶ Risk was higher for males and young adults and motorcycles were commonly involved. ▶ Males were much more likely to report drink driving than females. ▶ The prevalence of seat belt and helmet wearing was higher than previously reported. ▶ We will monitor changes in transport injury risk and related behaviour in this cohort.

## 1 Introduction

Traffic injuries are an important contributor to the national disease burdens of middle-income countries, and are estimated to cost 2% of the Gross Domestic Product (Roberts, 2004). The increasing death and disability from road trauma in middle-income countries can be largely attributed to massively increasing motorisation in the context of inadequate infrastructure, vehicular safety and safe system regulation (Peden et al., 2004). It can be viewed primarily as a development issue, as it is both a direct consequence of increasing industrialisation and modernisation and a huge constraint on development itself (Hyder and Ghaffar, 2004; McMahon and Ward, 2006; World Health Organization, 2009).

In Thailand, a middle-income “transitioning” country, there has been a dramatic upward trend in transport-related injuries and deaths that parallels rapid economic development and increasing motor vehicle ownership and use. The population injury rate from road traffic collisions has increased from 17 per 100,000 in 1984 to 152 per 100,000 in 2005 (Wibulpolprasert, 2008). Concurrently, traffic injury has become a leading cause of death in Thai males and females aged between 15 and 45 years, with over 5000 young adults dying each year. Road traffic injuries also cause the most permanent disability in Thailand (Sitthi-amorn et al., 2007).

A major barrier to addressing transport injury in low to middle-income countries is the lack of information about the nature and extent of non-fatal trauma and the prevalence of behavioural safety indicators and compliance with legislation, e.g. helmet and seat belt use and drink driving (Odero et al., 1997; WHO, 2009).

While Thailand has a stronger injury surveillance system than many middle-income countries, these issues still apply to the road safety data. Data on road traffic collisions in Thailand comes from three sources: hospital data which are collected intermittently from a small number of hospitals; police data which lack a standardised recording system and key information; and data from the Traffic Engineering Division, Department of Highways which cover only a quarter of national roads and largely rely on police reports (Suriyawongpaisal and Kanchanasut, 2003). The large community-based Thai National Injury Survey of 2003/2004 reported on over 300,000 Thais nationwide, however it recorded only injuries needing medical treatment and/or 3 days or more off work. It also relied on one respondent reporting for the whole household (Sitthi-amorn et al., 2007). Consequently, many minor injuries may have been missed.

The aim of this study is to determine the baseline frequency and distribution of transport injury and the prevalence of various road safety behaviours in a newly recruited cohort of Thai adults. This information is intended to serve as a baseline for monitoring changes in traffic crash risks and risk behaviours in response to ongoing implementation of policy and programs to address the problem of transport-related injury in Thailand.

## 2 Methods

### 2.1 Study design, setting and participants

The Thai Health-Risk Transition Study includes an ongoing Thai Cohort Study (TCS) of 87,134 adult Open University students residing across all regions of Thailand. The cohort comprises distance-learning students enrolled at Sukhothai Thammathirat Open University (STOU) which is referred to as an open university because it does not require high school graduates to pass an entrance test. The baseline TCS data were collected in 2005 and include information on transport injury and a wide array of demographic, socio-economic, behavioural and transportation factors that could be linked to Thailand's transport risks.

Details on population selection and methodology have been reported elsewhere (Sleigh et al., 2008). Briefly, the 2005 student register listed approximately 200,000 names and addresses: a 20-page questionnaire was mailed out to each student and 87,134 (44%) responded.

### 2.2 Measures

#### 2.2.1 Transport injury

All respondents were asked, ‘In the *last 12 months* how many injuries have you had that were serious enough to interfere with daily activities and/or required medical treatment?’ For their most serious injury, respondents were asked, ‘where were you when you were injured’ and ‘was this injury related to transport’. Location of the most serious injury was coded as home, road, sports facility, agricultural workplace, non-agricultural workplace or other. If the most serious injury was related to transport then we ascertained the respondent's role (driver, passenger, pedestrian), and, for drivers and passengers, the vehicle they were in (bicycle, motorbike, bus–van, car-pickup, other vehicle).

#### 2.2.2 Transport-related risk behaviours

We asked the entire cohort whether they had driven a motor vehicle after 3 or more glasses of alcohol in the previous 12 months. We categorised respondent use of safety devices for motorbikes (helmets) and cars (seat belts – back and front seats), as never, sometimes or always for the whole cohort.

### 2.3 Data management and analysis

Data scanning, verifying, and correcting were conducted using Scandevet, a program developed by a research team from Khon Kaen University. Further data editing was completed using SQL and SPSS software and for analyses we used SPSS and Stata. Descriptive data regarding the distribution of transport injury by sex, age and residence are presented. For those respondents that reported transport injury, their role and the vehicle involved in the injury event are presented by sex and age. Descriptive data regarding the age–sex distribution of transport-related risk behaviours for the whole cohort were also derived.

### 2.4 Ethical issues

Ethics approval was obtained from Sukhothai Thammathirat Open University Research and Development Institute (protocol 0522/10) and the Australian National University Human Research Ethics Committee (protocol 2004344). Free and informed written consent was obtained from all participants.

## 3 Results

### 3.1 Cohort description

The TCS cohort was 54.7% female, with a median age of 29 years. Compared to the overall STOU student population, the respondents were slightly older, but similar in terms of sex, education level, monthly income and geographic residence. In comparison to the Thai adult population, the cohort had a slightly higher proportion of females, a greater proportion of young to middle aged adults (21–40) and less in the younger (<21) and older (>40) age-groups. They were substantially more educated. Average monthly income compared to the Thai population is difficult to judge due to the large proportion that had no income or did not report their income in the Thai population survey. It appears that the TCS may be a little better off, however their average income is still quite modest. The geographic distribution of the cohort, however, was similar to the Thai population (Seubsman et al., in press) (Table 1).

### 3.2 Distribution and frequency of transport injury

**Fig. 1:**
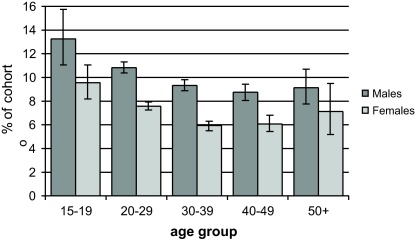
Frequency of transport injury over previous 12 months, by sex and age.

Overall 7279 (8.4% or 8354 per 100,000) of cohort respondents reported a transport-related injury as their most serious injury. In all age groups, males reported more transport injury than females. Young adults were more likely to report transport injury than older adults (Fig. 1). There was little difference in the proportion who reported transport injury between country residents (8.6%) and city/town residents (8.1%).

**Fig. 2:**
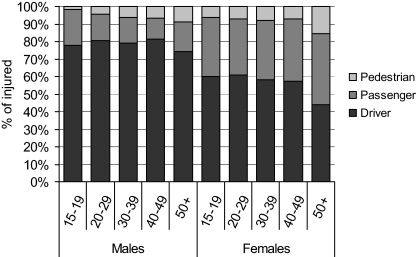
Role when injured, by sex and age.

Fig. 2 explores the roles the injured respondents played on the occasion of their transport-related injury. Of the 6371 injured persons who reported their role, there were 4514 (70.9%) drivers, 1459 (22.9%) passengers and 398 (6.2%) pedestrians. In all age groups, a higher proportion of injured males were likely to be drivers compared to injured females, with the reverse being true for passengers. A higher proportion of females (7.2%) experiencing transport-related injury were pedestrians compared to males (5.4%) and this injury category was the only one with a notable age-effect, with the proportion of injured that were pedestrians increasing with age for both sexes, reaching 15.6% among women over 50.

**Fig. 3:**
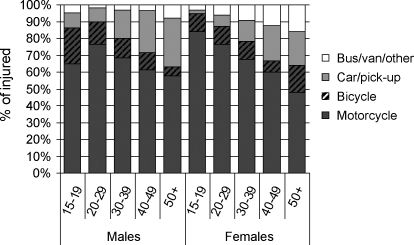
Vehicle used by drivers and passengers when injured, by sex and age.

Among the injured, 71.9% were riding motorcycles when their transport injury occurred (Fig. 3). This proportion decreased in older age groups but motorcycles were still the most common vehicle involved in transport injury events in all age groups in both sexes (70.8% for men, 73.2% for women). The proportion injured in cars or buses and vans increased with age for both sexes while the proportion injured while riding bicycles decreased with age for men but not for women. Injuries sustained while a driver or passenger in other types of vehicles (e.g. train, boat or airplane) were uncommon.

### 3.3 Prevalence and distribution of transport-related risk behaviours

**Fig. 4:**
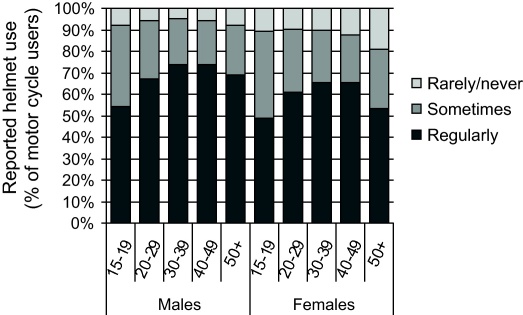
Reported helmet use for motorcycle drivers and passengers in the cohort, by sex and age (excluding 9685 who reported not riding motorbikes).

The distribution of transport-related risk behaviours was determined for the whole cohort, in order to better target interventions. Of the respondents who reported using a motorbike, approximately 6% of males and 10% of females rarely or never wore a helmet (Fig. 4). Overall, females were less likely to wear a helmet than males, with the highest proportion of non-wearers being women over 50 years of age (18.9%).

**Fig. 5:**
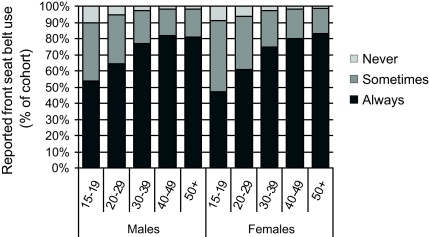
Reported front seat belt use for the cohort, by sex and age (excluding 1155 who said the vehicle does not have a front safety belt).

Of the respondents who had a front seat belt available for use in the vehicle, 72.2% of males and 66.6% of females reported always wearing the front seat belt and this increased with age (Fig. 5). Teenagers had the highest proportion of respondents who reported never wearing a front seatbelt (10% of males, 9% of females).

**Fig. 6:**
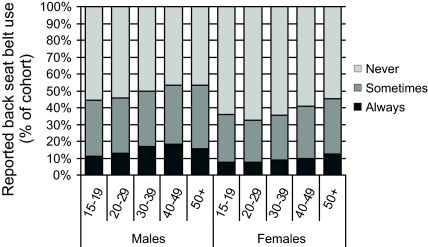
Reported back seat belt use for the cohort, by sex and age (excluding 23,813 who said the vehicle does not have a back safety belt).

In contrast, back seat belt use was low (Fig. 6). Of those who said the vehicle had a back seat belt available, over 50% of males and 65% of females never wore it, although as age increased there was a reduction in the proportion who never wore back seat belts. For all age-groups, men were more likely than women to sometimes or always wear the back seat belt.

**Fig. 7:**
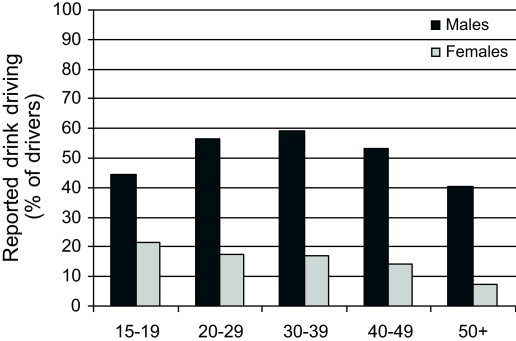
Reported drink driving during the previous 12 months for drivers in the cohort, by sex and age (excluding those who reported that they did not normally drive and those who reported never drinking alcohol).

Male drivers were much more likely to report having driven after 3 or more glasses of alcohol in the previous 12 months than female drivers (56.1% compared to 17.2%; Fig. 7). This comparison excluded respondents who said that they never drank alcohol (39.0% of females and 10.5% of males in the cohort).

## 4 Discussion

### 4.1 Transport Injury

In this study, 8.4% (8354 per 100,000) of the cohort reported that their most serious injury was transport-related, with males and teenagers more likely to be injured and motorcycles the most common vehicle involved. The proportion of injured who were pedestrians increased with age.

As anticipated, the transport injury rates were substantially higher than previously reported for Thailand. Estimates of 152 and 650 per 100,000 population were reported from the Thai Ministry of Public Health (Wibulpolprasert, 2008) and the community-based Thai National Injury Survey, respectively (Sitthi-amorn et al., 2007). The overall patterns of factors surrounding the transport injury, however, are similar with the young and males being the most at risk, motorcycles being the most common vehicle involved and the risk of being injured as a pedestrian increasing with age.

Both previous population surveys used higher injury severity thresholds than our study. The Ministry of Public Health data captured hospitalised injuries (Wibulpolprasert, 2008), while the National Injury Survey captured injuries requiring three or more days off work and/or medical attention (Sitthi-amorn et al., 2007). Further, the National Injury Survey relied on the head of the house reporting for all members, compared with our study which involved self-report. Because our TCS included minor injuries and the respondent reported on their own injuries, we would expect our injury estimates to be higher. Very minor injuries may still have been overlooked as respondents may not recall less serious injuries that occurred during the previous 12 months. In addition, we missed capturing data on transport injuries that were not the most serious injury that the respondent sustained. Thus our estimate is probably conservative.

The external validity of the study in terms of extrapolating the results to the Thai adult population is difficult to judge. Our cohort has slightly more females, less young (<21) and older (>40) adults and is more educated than the Thai adult population. Considering that young males are at higher risk of injury, and our cohort includes proportionally less of these than the Thai population, the estimates may be conservative. However, we also have proportionally less older adults, which may serve to increase the transport injury estimates. The higher education level of the respondents may also lead to a decreased injury rate relative to the general population. It is important to note however, that we are reporting the baseline data for an ongoing cohort study, so the real strength in these data lies in the internal validity for monitoring changes in this cohort over time.

### 4.2 Transport-related risk behaviours

The cohort reported a much higher prevalence of the use of safety devices (motorcycle helmets and seat belts) than previously reported for Thailand in the World Health Organization (WHO) Global Status Report on Road Safety (WHO, 2009). Almost two-thirds of motorcycle users (65%) reported always wearing a helmet compared to 27% in 2005. This rate is lower than for other Southeast Asian middle-income countries with similar legislation, e.g. Malaysia and Indonesia, that reported helmet-wearing rates of 90% or more in 2007 (WHO, 2009). Since helmet wearing was made mandatory in Thailand, however, enforcement has been maintained (WHO, 2009) and may be starting to have the desired cumulative effect. The lower helmet use rate reported by females was notable and could reflect concern with hair-style or hygiene but we did not gather data on motives.

Over two-thirds of respondents (69%) reported always using a front seat belt compared to 56% in 2005. This is similar to Malaysia in 2003, where the seat belt wearing rate was 70%, but less than Indonesia in 2005 when the rate was 85% (WHO, 2009). Only 11% reported always using a back seat belt, however this was still more than previous estimates (3% in 2005; WHO, 2009). There is substantial room for improvement if the Thai population is to reach the wearing rates evident in many of the high income countries (e.g. Australia, New Zealand and Germany in 2006/2007) of 95% or more for front seat belts and 87% or more for back seat belts (WHO, 2009). Considerable benefit would be achieved by mandating seat belt wearing for all vehicle occupants, rather than just front seat occupants as at present.

Excluding respondents that reported never drinking alcohol, more than half of the male drivers (56.1%) reported driving after three or more glasses of alcohol compared to 17.2% of female drivers. The high prevalence of drink driving among male Thai drivers has been reported previously with a cross-sectional study of blood alcohol (BAC) content in 4778 Thai drivers finding 8.7% with a blood alcohol content greater than 50 mg/dl (Chongsuvivatwong et al., 1999). Females were excluded from their analysis, however, because they only made up 2% of the sample. To our knowledge, ours is the first study to highlight the differences between men and women in terms of driving under the influence of alcohol in Thailand. Considering that female respondents in our cohort were much more likely to report never drinking at all (39.0% compared to 10% of males), and those that did drink alcohol were much less likely to drink drive than male drivers, it appears that drink driving countermeasures should be targeted heavily towards male drivers.

Our estimates of the prevalence of transport-related risk behaviours may be biased due to the self-report nature of data collection. Respondents may fail to report behaviours that are risky or socially unacceptable, however, many of our respondents did report drink driving or never wearing seat belts so this does not appear to be a major issue. The lower proportion of young adults aged under 21 and higher education level of our cohort compared to the Thai population may explain the higher estimates for helmet wearing and seat belt wearing than have been found previously. Consequently, the prevalence of the use of safety devices reported in this study probably represents the most optimistic estimates of current safety practices in Thailand. It is, however, unclear what effect this might have on the generalisability of estimates of the prevalence of drink driving. Again, however, we must emphasise the value of our data in terms of the internal validity for monitoring changes in this cohort over time.

### 4.3 Conclusions

We found much higher rates of transport injury than previously estimated by any other source, most likely due to the inclusion of less severe injuries. The findings give an overview for Thailand based on a large national sample of transport using adults and can assist with targeting the groups most at risk of transport injury (males, youth and motorcycle users), and the groups that display the most risky behaviour (drink driving for males, helmet wearing for females and back seat belt use for all age–sex strata). These data are an important addition to the evidence on which Thai authorities can base informed policies for addressing one of the major causes of individual and social burden of health in Thailand. The study goes some way towards redressing the data deficiencies identified by the WHO as one of the major barriers to addressing the problem of transport injury in lower and middle income countries (WHO, 2009). Finally, and perhaps of most value, our cohort study provides an opportunity to monitor changes in these risks and behaviours in the same cohort into the future.

## Conflict of interests

The authors have no competing interests.

## Funding

This study was supported by the International Collaborative Research Grants Scheme with joint grants from the Wellcome Trust UK (GR071587MA) and the Australian NHMRC (268055). The funding sources played no role in study design, data collection, analysis or interpretation, writing the report, or the decision to submit the paper for publication.

## Figures and Tables

**Table 1:** TCS cohort characteristics: comparison with STOU student body and Thai population in 2005 (adapted from Seubsman et al., in press).

Distribution (%)	TCS cohort	aSTOU	bThai population >15 years	
*Demographic characteristics*				
Sex				
Male	45.3	46.8	49.5	
Female	54.7	53.2	50.5	
Age (years)				
<21	6.2	16.3	13.3	
21–30	51.5	56.2	23.9	
31–40	29.3	19.6	21.6	
41–50	11.0	6.7	17.5	
>50	2.0	1.2	24.7	
*Socioeconomic status*				
Education (highest completed)				
Lower than junior high school			56.6	
Junior to high school	48.9	44.9	30.9	
Diploma	27.0	32.7	3.7	
University	24.2	22.4	8.4	
Monthly income (Baht/month)^c^				Monthly income (Baht/month)
			*41.6*	*No income/not reported*
≤3000	11.0	14.7	*18.9*	≤*3000*
3001–7000	30.9	23.9	*19.7*	*3001–6000*
7001–10,000	23.3	24.7	*7.6*	*6001–9000*
>10,000	34.7	36.8	*12.2*	>*9000*
*Geographic residence*				
Bangkok and Central	41.4	40.8	37.1	
North	18.1	18.1	18.6	
East and Northeast	26.8	25.7	32.0	
South	13.0	15.1	12.3	

a Data from STOU annual report, 2005.

b Thai Health and Welfare survey, 2005.

c Income categories available were slightly different for the Thai population compared to TCS and STOU.
